# Leveraging Machine Learning Classifiers in Transfer Learning for Few-Shot Modulation Recognition

**DOI:** 10.3390/s26020674

**Published:** 2026-01-20

**Authors:** Song Li, Yong Wang, Jun Xiong, Xia Wang

**Affiliations:** Beijing Institute of Tracking and Telecommunications Technology, Beijing 100094, China

**Keywords:** deep learning, few-shot learning, machine learning, modulation recognition, transfer learning

## Abstract

The rapid advancement of communication systems has heightened the demand for efficient and robust modulation recognition. Conventional deep learning-based methods, however, often struggle in practical few-shot scenarios where acquiring sufficient labeled training data is prohibitive. To bridge this gap, this paper proposes a hybrid transfer learning (HTL) approach that synergistically combines the representation power of deep feature extraction with the flexibility and stability of traditional machine learning (ML) classifiers. The proposed method capitalizes on knowledge transferred from large-scale auxiliary datasets through pre-training, followed by few-shot adaptation using simple ML classifiers. Multiple classical ML classifiers are incorporated and evaluated within the HTL framework for few-shot modulation recognition (FSMR). Comprehensive experiments demonstrate that HTL consistently outperforms existing baseline methods in such data-scarce settings. Furthermore, a detailed analysis of several key parameters is conducted to assess their impact on performance and to inform deployment in practical environments. Notably, the results indicate that the K-nearest neighbor classifier, owing to its instance-based and non-parametric nature, delivers the most robust and generalizable performance within the HTL paradigm, offering a promising solution for reliable FSMR in real-world applications.

## 1. Introduction

In wireless communication systems, modulation recognition serves as a core technology capable of real-time analysis of non-cooperative signal modulation characteristics and accurate determination of their modulation types [1]. This technology has demonstrated broad application potential in critical fields such as cognitive radio and dynamic spectrum access, as well as radio monitoring and spectrum management [2]. In the early stages of radio technology development, the electromagnetic environment was relatively ideal, and signal types were limited. As a result, modulation recognition could be achieved through manual observation of frequency-domain spectral lines and time-domain waveforms, provided that expertise in the communication domain was available. Recently, with the continuous evolution of 5G/6G communications [3,4], the Internet of Things [5], and integrated space–air–ground networks [6], the demand for efficient and robust modulation recognition techniques is growing increasingly urgent.

### 1.1. Related Works and Motivations

Typical modulation recognition comprises a three-stage pipeline consisting of data preprocessing, feature selection, and classification. In the data preprocessing stage, operations such as outlier handling and data type conversion are implemented on the received signal to facilitate subsequent feature selection [7]. In the feature selection stage, intermediate representations of the signal, such as features, sequences, or images, are used to obtain discriminative characteristics. The representations are derived from the raw signal, including, but not limited to, cumulant [8] and circular [9] features. The sequence representation includes the raw in-phase/quadrature (I/Q) sequences [10], and other transformed sequences such as the amplitude, phase, and frequency sequences, along with their various combinations [11]. The image representation is formed by two primary types: constellation diagrams [12] and time–frequency images [13]. In the classification stage, traditional machine learning (ML) classifiers, for example, K-nearest neighbors (KNN) [14] and support vector machine (SVM) [15], were extensively employed. These methods, however, often demand considerable expertise and effort for manual feature crafting and parameter calibration, a process that is both time-consuming and susceptible to suboptimal outcomes. In contrast, deep learning (DL) involves two primary components in the classification stage: a feature extractor and a classifier. The former is responsible for extracting more discriminative high-dimensional features, while the latter operates on these features for final classification. From the network architecture perspective, the feature extractor is often called a backbone network, while the classifier is often called a classification head. These two components are trained end-to-end, without the need for manual feature engineering.

The rise of DL has catalyzed a paradigm shift in modulation recognition, establishing DL-based methods as a dominant approach. Owing to their raw format, I/Q sequences are frequently employed as inputs to DL models [16,17,18]. This approach avoids explicit signal transformation, thereby saving computational time and removing the need for manual parameter tuning. The convolutional neural network (CNN) architecture, proven successful in computer vision, has also been applied to modulation recognition, leveraging its strengths in spatial feature extraction. Specifically, the CNN in [10], despite its simple few-layer architecture, demonstrated superior accuracy over traditional ML classifiers. Moreover, the residual network (ResNet) [19], originally developed for image classification, has been appropriately adapted to handle the modulation recognition task [20]. Comparative analysis confirms that ResNet outperforms simple CNNs in classification accuracy while requiring fewer parameters. Furthermore, by designing specific convolutional blocks, an efficient CNN architecture named MCNet was proposed in [21], achieving even higher accuracy. The applicability of successful DL architectures has extended beyond their original domains; models such as recurrent neural networks (RNNs) [22], including the long short-term memory (LSTM) [23] and gated recurrent unit (GRU) [24] variants, along with Transformers [25,26], prominent in natural language processing, have found effective use in modulation recognition. Hybrid models combining the aforementioned DL architectures have also been explored [27,28]. However, conventional DL methods suffer from an inherent limitation: they require a large amount of labeled training data to achieve high accuracy, which is often unavailable in practical scenarios, especially in non-cooperative situations. Driven by the need to learn effectively from scarce data, few-shot learning (FSL) has drawn significant research interest.

The core objective of FSL is to learn a model that can achieve high classification accuracy with only a few training samples per class [29]. In recent years, numerous FSL methods have been proposed. These approaches can be broadly divided into three categories. The first category of FSL methods focuses on improving data utilization, typically through data augmentation techniques. For example, flipping, truncation, and rotation operations can be applied to I/Q sequences to effectively augment the existing few-shot dataset and enhance model generalization [30,31]. Consequently, standard network architectures, including CNN, ResNet, and the other advanced models mentioned above, are readily applicable in few-shot settings. Semi-supervised learning techniques that combine both labeled and unlabeled data have also been explored. This can somewhat reduce the need for labeled data while still leveraging the unlabeled data to improve model generalization. Specifically, a hybrid model using two parallel auto-encoders for label-agnostic feature extraction was proposed in [32], making it naturally suitable for semi-supervised scenarios. In addition, a deep residual shrinkage network [33] designed for this learning setting was explored. However, when the available samples are severely limited (i.e., in cases of acute scarcity), the effectiveness of such approaches is limited. The second category of FSL methods focuses on improving model architecture through deliberate design informed by expertise. For example, the capsule network [34] was introduced to better preserve the spatial relationships within signals through its specialized capsule layers. Moreover, a modular FSL framework named MsmcNet [35], which integrates dedicated signal processing modules for high-level feature extraction, was also investigated. Alternatively, the few-shot classification problem can be addressed within a hybrid inference framework. For example, this can be achieved by designing a two-channel CNN and a temporal convolutional network to extract spatial and temporal features in parallel, followed by measuring sample similarity in these dual feature spaces [36]. Similarly, by combining a convolutional layer, residual modules, multi-component down sampling modules with a fully connected (FC) layer, the multi-component extraction network was proposed in [37]. In addition, the multi-scale feature fusion and distribution similarity network [38] that can effectively extract features across different receptive fields was also studied. These methods show satisfactory classification performance in few-shot modulation recognition (FSMR) tasks. However, the reliance of these methods on particular network architectures may hinder generalization across diverse tasks. The third category of FSL methods focuses on learning transferable knowledge (or representations) through the paradigm of transfer learning (TL). By distilling generalizable knowledge from class-disjoint auxiliary datasets, TL enables rapid adaptation to novel tasks with limited samples [39]. It can effectively bridge the data scarcity gap and thus has emerged as a promising alternative in the FSL domain. The efficacy of TL for FSMR has been established by several studies [40,41,42], which report high accuracy with few samples per class, surpassing traditional approaches. Different from TL, meta-learning follows a “learning-to-learn” paradigm. It trains a model by constructing numerous few-shot tasks (episodes) from the auxiliary dataset to simulate the target few-shot scenario [43]. Two representative meta-learning methods, the prototypical network [44] and relation network [45], have attained notable accuracy in FSMR tasks. In this type of approach, advanced network architectures can serve as the backbone for feature learning. At its core, both TL and meta-learning operate on the principle of leveraging pre-trained models to circumvent the need for training from scratch (TFS). This enables the training process to be substantially simplified by leveraging informed prior knowledge rather than beginning from a completely blank slate. This study is primarily centered on the TL paradigm, yet remains readily extensible to the meta-learning paradigm. Our analysis will reveal that despite TL’s ability to reduce sample dependence, the one- or few-shot scenario presents a persistent and severe challenge. The underlying issue may lie in the insufficiency of data provided during the fine-tuning stage of TL to properly train the classifier network, resulting in suboptimal performance in few-shot settings. Unlike prior works that primarily employ dedicated networks as backbones to extract distinctive features, this study shifts focus towards enhancing classifier performance, placing less emphasis on the feature extractor itself.

### 1.2. Contributions and Organization

To overcome the practical limitations of conventional TL, this paper introduces a hybrid transfer learning (HTL) approach that synergistically integrates TL with ML, thereby marrying the representation power of DL to the robustness of ML. The HTL approach operates in two stages. In the first stage, TL is employed to obtain an effective feature extractor, thereby retaining DL’s advantage of automated feature extraction. In the second stage, the extracted deep features are used to train a traditional ML classifier, which preserves ML’s benefits of rapid, non-iterative training and robust few-shot performance. The main contributions of this work are summarized as follows:

- We propose to incorporate traditional ML classifiers within the TL framework to achieve high classification accuracy in FSMR tasks.
- We validate the proposed method through a series of experiments, with comparisons to conventional TFS and TL approaches.
- We examine several parameters that may influence practical performance, aiming to assess their impact on classification accuracy.
- We investigate the integration of HTL with meta-learning paradigms, establishing its effectiveness and transferability.

The remainder of this paper is structured as follows. Section 2 presents the signal model along with its associated data preprocessing steps, and formulates the FSMR problem. Section 3 reviews the foundational TFS and TL approaches, and proposes the HTL approach incorporating an introduction to the specific ML classifiers. Section 4 introduces the backbone network to extract discriminative features for classification. Section 5 describes the dataset used in this study, and provides experimental results and analysis. Finally, Section 6 concludes this paper and discusses future work.

## 2. Preliminaries

This section outlines the theoretical foundations of modulation recognition. First, a mathematical model of wireless signals is established, and complex-valued signals are converted into a three-dimensional (3D) tensor format for DL processing. Subsequently, the modulation recognition problem is mathematically formulated as a multi-class classification task, and the definition of few-shot recognition is illustrated.

### 2.1. Signal Model and Data Preprocessing

In wireless communication systems, signals typically propagate through complex communication channels before being captured by the receiver. The received signal at the receiver end, which contains both the desired signal and other channel-induced impairments, can be mathematically expressed as

(1) $$\begin{array}{c} x\left(t\right)=s\left(t\right)*h\left(t\right)\cdot e^{j(2\pi f_{d}t+\varphi )}+\eta \left(t\right) \end{array}$$

where ∗ denotes convolution, s(t) is the baseband modulating signal, h(t) is the equivalent baseband channel impulse response, $f_{d}$ is the Doppler frequency, φ is the initial carrier phase, and η(t) is the complex-valued noise.

Then, after discrete sampling, the received signal can be represented in the I/Q format, given by

(2) $$\begin{array}{c} x\left[l\right]=x_{I}\left[l\right]+jx_{Q}\left[l\right],\;\;l=1,2,\ldots ,L \end{array}$$

where $x_{I}\left[l\right]=ℜ\left\{x(l)\right\}$, $x_{Q}\left[l\right]=ℑ\left\{x(l)\right\}$, and ℜ(·) and ℑ(·) denote the real and imaginary parts, respectively.

To maintain compatibility with mainstream DL techniques that require 3D tensor inputs, this study adopts a straightforward time-domain data preprocessing method.

First, the received sampling points from Equation (2) are organized into an I/Q sample matrix, given by

(3) $$\begin{array}{c} X=\left[\begin{array}{c} x_{I}\left[1\right],x_{I}\left[2\right],\ldots ,x_{I}\left[L\right]\\ x_{Q}\left[1\right],x_{Q}\left[2\right],\ldots ,x_{Q}\left[L\right] \end{array}\right]. \end{array}$$

Thus, the raw signal with *L* complex I/Q samples is transformed into a real-valued representation to facilitate processing.

**Figure 1:**
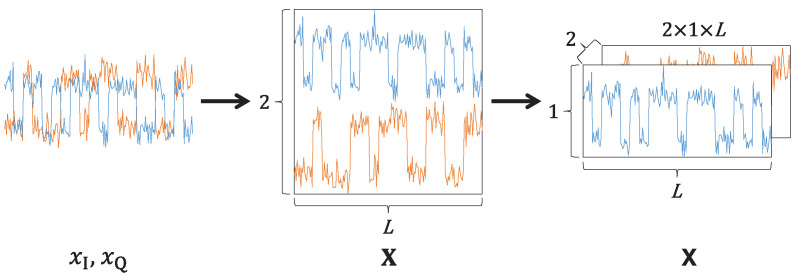
Schematic of data preprocessing.

Subsequently, the input X are converted into a 3D tensor X, with dimensions such as 2×1×L. Here, “2” denotes the channel dimension, which represents the I/Q components. Moreover, 1×L represents the spatial dimensions, which means a height of 1 and a width of *L*. This method converts the raw input into a unified format, facilitating direct and efficient processing with both one-dimensional (1D) and two-dimensional (2D) neural network architectures. The overall data preprocessing procedure is visually shown in Figure 1.

### 2.2. Few-Shot Modulation Recognition (FSMR)

The objective of modulation recognition is to identify the modulation scheme of the baseband signal s(t) in Equation (1). Since the signal waveform retains modulation information, X provides an effective representation for modulation recognition.

From a maximum a posteriori standpoint, the modulation recognition problem can be seen as a multi-class classification problem:

(4) $$\begin{array}{c} \hat{y}=arg maxp\left(y|X\right) \end{array}$$

where $\hat{y}$ is the predicted label, *y* is the true label, and p(y|X) is the probability of possible class labels given X.

To handle the unknown p(·) in Equation (4), it is often approximated in DL approaches by a mapping function, making the problem become

(5) $$\begin{array}{c} \hat{y}=arg maxf\left(X;W\right) \end{array}$$

where f(·) is the mapping function (model) with parameters W that outputs a probability vector associated with *y*. Therefore, the key objective becomes constructing f(·) with suitable W to map the sample space X to the class space Y.

In real-world applications, the FSMR problem is often encountered, owing to the challenge of acquiring sufficient high-quality signal samples for training. “Few-shot” means that the training dataset, denoted as $D_{\mathrm{fs}}={\left\{\left(X_{i},y_{i}\right)\right\}}_{i=1}^{N_{\mathrm{fs}}}$, contains a small amount of supervised samples. Typically, it contains *N* classes with *K* samples per class, and $N_{\mathrm{fs}}=N\times K$ is the number of training samples. *K* is relatively small, usually no greater than 20. This kind of few-shot classification task is called an *N*-way *K*-shot task.

## 3. Methodologies

This section provides a detailed exposition of several approaches for FSMR, along with a comparative analysis. First, TFS, as a basic DL method, is described, highlighting its susceptibility to overfitting in data-scarce settings. Subsequently, TL is introduced as a two-stage strategy comprising pre-training and fine-tuning, designed to mitigate this issue. Finally, we present the HTL approach that integrates deep feature extraction with traditional ML classifiers, further enhancing both performance and flexibility under few-shot conditions. Through systematic comparison of these methodologies, their distinctive characteristics are clarified in detail.

### 3.1. Training from Scratch (TFS)

In DL approaches, f(·) is designed as a neural network with multiple layers of non-linear transformations. The last layer contains *N* neurons, each of which outputs the probability of the candidate class label. The predicted label is the one with the highest probability according to Equation (5).

On this basis, W can be optimized by training the network with training samples. To derive W, TFS has become the predominant and well-established method. Specifically, DL algorithms learn the parameters W in Equation (5) using mini-batch gradient descent over numerous iterations, as follows:

(6) $$\begin{array}{c} W^{\left(k+1\right)}=W^{\left(k\right)}-\eta \nabla_{W^{\left(k\right)}}\left[\frac{1}{\left|B\right|}\sum_{i\in B}L\left(f\left(X_{i};W^{\left(k\right)}\right),y_{i}\right)\right] \end{array}$$

where *k* is the iteration index, *B* is the index set of the batch of samples, |B| is the batch size, η is the learning rate, and L(·) is the loss function, which is usually the cross-entropy loss in classification tasks. The entire training dataset is divided into multiple mini-batches; a complete and non-repeating traversal of these mini-batches constitutes an epoch.

By using a large number of training samples, TFS can obtain a suitable $W^{*}$ and thus achieve high classification accuracy. However, without enough training samples, it often easily leads to model overfitting when dealing with the few-shot recognition problem. This means that the model can only generalize well to the given training dataset, but not to the unseen test data.

### 3.2. Transfer Learning (TL)

In response to the few-shot challenge faced by TFS, TL has been a promising approach to address the model overfitting problem. It is designed to train the model on the auxiliary dataset first and transfer the learned knowledge to the target problem.

Typically, the model f(·) is divided into two cascaded components: a feature extractor (backbone network) and a classifier (classification head), and can thus be decomposed as

(7) $$\begin{array}{c} f\left(X;W\right)=f_{\mathrm{cl}}\left(f_{\mathrm{FE}}\left(X;W_{\mathrm{FE}}\right);W_{\mathrm{cl}}\right) \end{array}$$

where $f_{\mathrm{FE}}\left(\cdot \right)$ and $W_{\mathrm{FE}}$ are the feature extractor and its parameters, respectively, and $f_{\mathrm{cl}}\left(\cdot \right)$ and $W_{\mathrm{cl}}$ are the classifier and its parameters, respectively. In the DL domain, the feature extractor has multiple layers that can transform the input X into a high-level representation, ultimately producing the feature representation $f_{\mathrm{FE}}\left(X;W_{\mathrm{FE}}\right)$. In contrast, the classifier is usually a simple FC layer with the softmax activation function that maps the feature representation to the label probabilities.

TL involves the following two stages:

Stage 1: First, the feature extractor is usually pre-trained on a sufficiently large auxiliary dataset $D_{\mathrm{au}}={\left\{\left(X_{a},y_{a}\right)\right\}}_{a=1}^{N_{\mathrm{au}}}$ with TFS. This is called the pre-training stage, and it can be implemented by replacing $D_{\mathrm{fs}}$ with $D_{\mathrm{au}}$ and using Equation (6). Thus, a suitable $W_{\mathrm{FE}}^{*}$ for the feature extractor can be obtained. Note that $D_{\mathrm{au}}$ is typically collected from a different source than $D_{\mathrm{fs}}$, which means $D_{\mathrm{fs}}\cap D_{\mathrm{au}}=⌀$, yet the two are assumed to share the same feature space and data distribution.

Stage 2: Next, TL freezes the feature extractor parameters $W_{\mathrm{FE}}^{*}$ obtained by TFS on $D_{\mathrm{au}}$ to preserve the general representations it has learned. Then, the fine-tuning stage is conducted to retrain the task-specific classifier using $D_{\mathrm{fs}}$, thereby obtaining a suitable $W_{\mathrm{cl}}^{*}$ by iterations similar to Equation (6), as follows:

(8) $$\begin{array}{c} W_{\mathrm{cl}}^{\left(k+1\right)}=W_{\mathrm{cl}}^{\left(k\right)}-\eta^{\prime }\nabla_{W_{\mathrm{cl}}^{\left(k\right)}}\left[\frac{1}{|B^{\prime }|}\sum_{i\in B^{\prime }}L\left(f_{\mathrm{cl}}\left(f_{\mathrm{FE}}\left(X_{i};W_{\mathrm{FE}}^{*}\right);W_{\mathrm{cl}}^{\left(k\right)}\right),y_{i}\right)\right] \end{array}$$

where $\eta^{\prime }$ is the learning rate, $B^{\prime }$ is the index set of the batch, and $|B^{\prime }|$ is the batch size, all in the fine-tuning stage.

Different from TFS (see Equation (6)), the fine-tuning technique employed by TL (see Equation (8)) optimizes only the simple classifier parameters $W_{\mathrm{cl}}$, with the feature extractor parameters $W_{\mathrm{FE}}^{*}$ fixed. By reducing the number of parameters that need optimization, this approach mitigates overfitting in TFS. Nonetheless, our experience shows that fine-tuning remains particularly challenging with an extremely limited number of training samples.

### 3.3. Hybrid Transfer Learning (HTL)

Considering that the classifier used in TL is a fixed-structure FC layer that may not be trained well with a small number of training samples, we aim to replace it with a more flexible ML classifier. Thus, the proposed HTL approach hybridizes a DL-based feature extractor with traditional ML classifiers.

HTL follows a similar two-stage framework to TL:

Stage 1: The first stage is identical to the TL approach in Section 3.2. That is, the feature extractor is pre-trained on $D_{\mathrm{au}}$ using TFS, then applied to $D_{\mathrm{fs}}$ to produce classifier input features: $z_{i}=f_{\mathrm{FE}}\left(X_{i};W_{\mathrm{FE}}^{*}\right)$.

Stage 2: The second stage is to obtain the ML classifier by fitting a function that maps input features to class labels. Its implementation involves solving an empirical risk minimization problem:

(9) $$\begin{array}{c} \min_{g\in G,\theta \in \Omega }\frac{1}{N_{\mathrm{fs}}}\sum_{i=1}^{N_{\mathrm{fs}}}L\left(g\left(z_{i};\theta \right),y_{i}\right) \end{array}$$

where G is the function space of the ML classifiers, Ω is the parameter space of the classifiers, g(·) is the ML classifier, and θ are the parameters. It is worth noting that, for KNN, the set of stored training samples $D_{\mathrm{KNN}}={\left\{\left(z_{i},y_{i}\right)\right\}}_{i=1}^{N_{\mathrm{fs}}}$ essentially serves as the model’s implicit parameters. Correspondingly, the KNN model can be expressed as $g_{\mathrm{KNN}}\left(\cdot ;D_{\mathrm{KNN}}\right)$. Therefore, the “optimization” or model construction step reduces to simply storing $D_{\mathrm{KNN}}$, with no iterative parameter update over θ required.

We focus on four representative ML classifiers: KNN, SVM, decision tree (DT), and random forest (RF). For implementation details, the reader is directed to the standard textbook [46]. Their basic principles and optimization characteristics are summarized as follows:

- KNN: Given a test sample, its *k* nearest neighbors are retrieved from the training data. The sample is then designated as the majority class among these neighbors. No explicit optimization: “Training” is merely storing data; the parameter space is fixed upon training.
- SVM: Its primary goal is to find the maximum-margin hyperplane that best separates the classes. Key techniques include the kernel trick for non-linear problems and soft margin for handling misclassifications. Convex optimization: Finds the maximum-margin hyperplane by solving a convex quadratic programming problem, guaranteeing a global optimum.
- DT: It builds a model by recursively splitting the dataset using the most discriminative features and thresholds. The goal is to create purer subsets, and predictions are the majority class or the mean value at the leaves. Greedy, discrete search: Employs a recursive, locally optimal splitting strategy with no guarantee of a globally optimal tree.
- RF: It constructs an ensemble of DTs. Each tree is trained on a random bootstrap sample of the data and a random subset of features. The final prediction is determined by majority voting. Parallel and independent greedy searches: Builds multiple diverse trees via Bagging and random feature selection, reducing the variance of the optimization process.

The parameter differences of these ML classifiers are summarized in Table 1. Traditional ML classifiers such as KNN, SVM, DT, and RF can adaptively form more complex and locally tailored decision boundaries through their unique mechanisms (e.g., local computation, the kernel trick, rule learning, and model ensembling). Consequently, in terms of structural flexibility, these algorithms surpass the FC classifier, which has a fixed parametric form.

### 3.4. Schematic Diagrams

**Figure 2:**
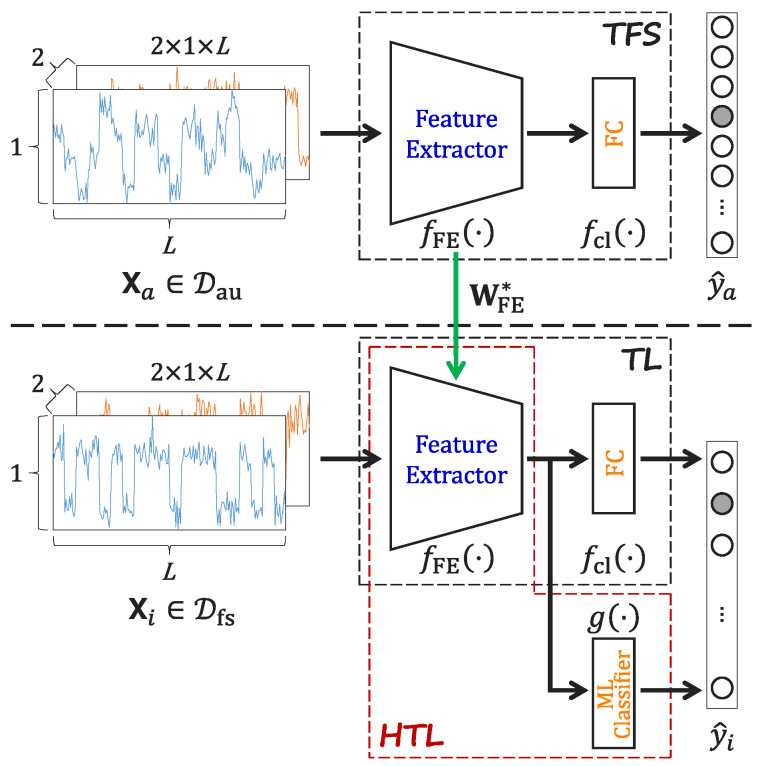
Schematic diagram of TFS, TL, and HTL.

To visualize the differences between TFS, TL, and HTL, a schematic diagram is presented in Figure 2. The figure illustrates the key components and training process of each method. The main differences between these methods are as follows:

- In the TFS framework, all model parameters are initialized completely randomly, followed by a complete, end-to-end training on the dataset without utilizing any external pre-trained knowledge.
- TL and HTL leverage pre-trained knowledge from a large-scale auxiliary dataset for the target few-shot task, initializing the feature extractor with proven parameters in contrast to a random initialization.
- HTL, in addition to the feature extractor, incorporates an ML classifier to perform the final classification task, which is different from the FC classifier used in both TFS and TL.

## 4. Backbone Network

The training procedures for the compared approaches have been detailed in Section 3, and their straightforward test steps can be implemented following Equation (5). Although these methods are not bound to a specific backbone architecture, describing the common network used is necessary for clarity and fair comparison.

**Figure 3:**
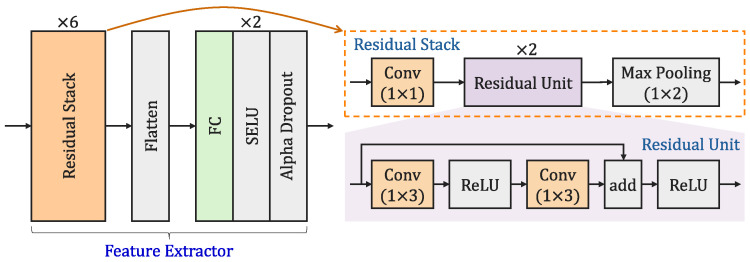
Structure of the backbone network.

We use ResNet [20] as the backbone network, a choice justified by its well-established efficacy in enabling stable deep network training and its proven performance in modulation recognition. Figure 3 illustrates the detailed structure of the backbone network. As shown on the left side of the figure, the backbone network employs a lightweight 1D ResNet for feature extraction. The network begins with six residual stacks, which progressively construct hierarchical feature maps. The feature maps produced by the residual stacks are spatially collapsed into a 1D vector via flattening, enabling subsequent processing. Next, the flattened output is progressively compressed and integrated across two sequential blocks, thereby retaining the most discriminative features. Each block is composed of an FC layer followed by a scaled exponential linear unit (SELU) activation function and an alpha dropout layer [47]. A detailed description of the residual stack and residual unit is provided below:

- Residual Stack: Each residual stack, as shown on the upper-right side of Figure 3, is composed of a 1×1 convolutional layer followed by two residual units, with a final 1×2 max-pooling layer. As the feature maps propagate through each residual stack, 1×1 convolution facilitates cross-channel feature interaction, while parameter-free pooling reduces the temporal dimension by half. This strategy not only exponentially expands the receptive field of subsequent layers but also suppresses high-frequency noise through pooling, thereby achieving deep and wide temporal encoding.
- Residual Unit: Each residual unit, as shown on the lower-right side of Figure 3, employs a skip connection. Its main path extracts temporal features via a cascaded 1×3 convolutional layer with rectified linear unit (ReLU) activations, while the identity branch provides a direct gradient pathway. This design alleviates vanishing gradients and preserves signal details. Finally, a ReLU activation is applied to the aggregated features from both paths, performing a sparsifying truncation that suppresses noise while enhancing representative capacity.

Table 2 summarizes the output dimensional structure of each layer in the backbone network. The network employs residual stacks as its core feature extraction module, achieving hierarchical transformation of feature dimensions through progressive downsampling. The input data dimension is 2×1×1024, representing a time-domain signal of 1024 sampling points with separate I and Q channels. After processing through six residual stacks, the feature dimension is progressively reduced from 32×1×512 to 32×1×16, enabling multi-scale abstract representation of the original signal. Finally, the network maps the high-dimensional features into a 128-dimensional feature space via two FC layers with SELU activation, providing a compact and discriminative feature representation for subsequent classification tasks.

## 5. Experimental Results and Analysis

This section presents extensive experiments and analyses to validate the performance of the proposed method. The experiments are organized into four parts. First, the dataset and parameter settings are introduced to establish the experimental foundation. Second, a comprehensive performance evaluation of different methods is conducted through feature visualization and comparative analysis. Third, the impact of key parameters on classification performance is analyzed to provide insights into method robustness. Finally, the method is validated on alternative learning frameworks and datasets to confirm its generalizability.

The comparative approaches, as illustrated in Section 3, include several FSL paradigms:

- TFS, which directly trains the network on the few-shot dataset.
- TL, where the last FC layer of a pre-trained model (obtained from the auxiliary dataset) is replaced by a new layer that is retrained on the few-shot dataset.
- The proposed HTL, which leverages the feature extractor from a model pre-trained on the auxiliary dataset, processes the few-shot dataset to generate feature representations, and then trains an ML classifier on these features. Specifically, four widely-used ML classifiers are implemented: KNN, SVM, DT, and RF [46].

### 5.1. Dataset and Parameter Settings

The experimental data used in this study are sourced from RadioML 2018.01A [20], a publicly available dataset widely adopted in the modulation recognition domain. This dataset encompasses 24 distinct modulation schemes, comprehensively covering a range from analog modulations such as AM-DSB and FM to digital modulations including OOK, BPSK, QPSK, and even high-order 256QAM. To approximate real-world channel conditions, the signal generation process incorporates not only additive white Gaussian noise but also a series of critical channel impairments, including carrier frequency offset, sampling rate offset, and fading effects.

Unless otherwise specified, the datasets and parameters used in the experiments are shown in Table 3. Each modulation class comprises 4096 samples per signal-to-noise ratio (SNR), with each sample comprising 1024 I/Q sampling points. The auxiliary dataset, used for model pre-training, comprises 12 modulation classes. Consequently, the auxiliary dataset contains a total of 65,536 samples per class, spanning the SNR range from −10 to 20 dB in 2 dB increments. The selection of −10 dB as the lower SNR boundary is primarily motivated by the observation that feature extractors encounter significant challenges in deriving discriminative features suitable for classification at lower SNR ratios, where the signal characteristics become increasingly obscured by noise interference. In the pre-training stage, the auxiliary dataset is partitioned into training and test sets in an 8:2 ratio, on which the model is trained to perform the classification task, learning generalizable feature representations from the data. The few-shot dataset contains five novel modulation classes not present in the auxiliary dataset, and is used for few-shot training and evaluation. During the training stage, only five randomly selected samples per class are used for model training or fine-tuning. During the testing stage, one sample per class per SNR not seen in the training stage is utilized for inference to evaluate the classification accuracy across various SNRs. Owing to the limited samples per experiment, the classification accuracy is estimated via 500 Monte Carlo trials.

In the experiments, all comparative methods are implemented in Python 3.13.3, with DL components developed using PyTorch 2.7.0 and ML components built with scikit-learn 1.6.1. To train the network, the cross-entropy loss function is employed. Model weights are optimized using the adaptive moment estimation (Adam) optimizer [48] with an initial learning rate of 0.001. A step-decay learning rate schedule is applied, reducing the learning rate to 0.8 times its previous value every 10 epochs. The training is conducted for 50 epochs with a batch size of 1024. The ML classifiers rely on either closed-form solutions or heuristic algorithms for optimization. Table 4 presents the hyperparameters used by the ML classifiers in the experiments, which are explicitly set values rather than default configurations. Unspecified parameters in the table remained at their scikit-learn defaults. It should be noted that hyperparameter tuning for DT and RF presents a greater challenge given their expansive parameter spaces. Conversely, SVM and KNN are simpler to optimize as they require adjusting only a few key parameters. For detailed explanations of parameter meanings and their functional roles, readers are referred to the scikit-learn official documentation [49].

### 5.2. Performance Evaluation of Different Methods

This subsection evaluates the performance differences between the proposed HTL approach and conventional methods through multi-dimensional experiments. First, the feature representation capabilities of different methods are qualitatively compared using t-distributed stochastic neighbor embedding (t-SNE) visualization [50]. Building on this, recognition performance is quantitatively evaluated based on classification accuracy. This is followed by a confusion matrix analysis to examine inter-class error patterns in depth, concluding with an assessment of computational efficiency.

#### 5.2.1. Visualization of Feature Representations

First, to assess the capacity of the feature extractors constructed by TFS and TL-based methods, the distribution of feature representations is evaluated. Specifically, these representations are non-linearly projected into a 2D plane using the widely adopted t-SNE algorithm [50].

**Figure 4:**
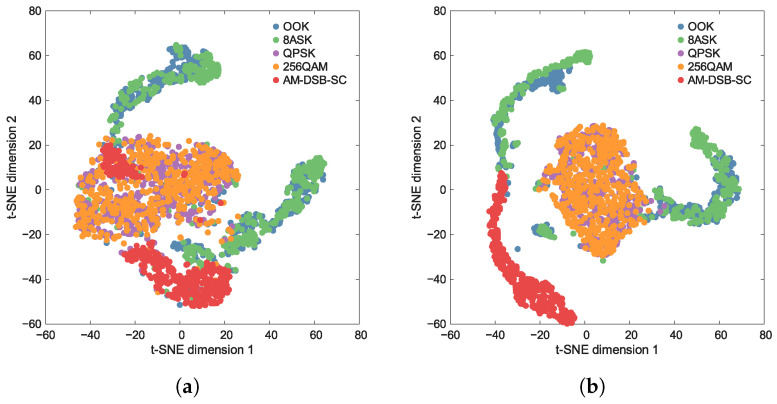
Distribution of feature representations at various SNRs obtained by using TFS. (**a**) SNR=−2 dB. (**b**) SNR=6 dB.

**Figure 5:**
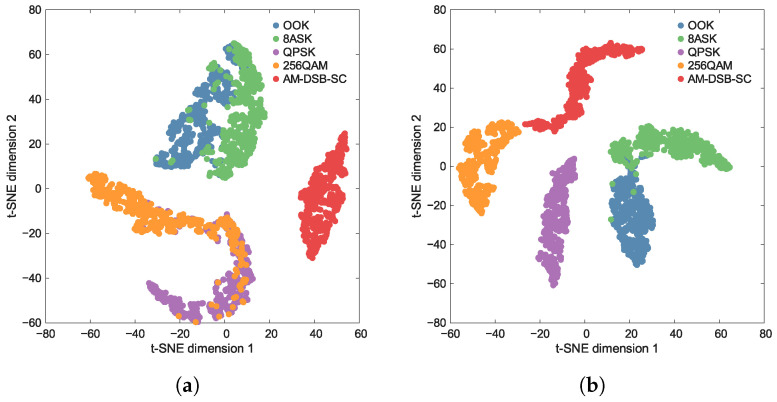
Distribution of feature representations at various SNRs obtained by using TL or HTL. (**a**) SNR=−2 dB. (**b**) SNR=6 dB.

Figure 4 and Figure 5 show the t-SNE visualizations at two different SNRs. By observing the distribution structure of the dimensionality-reduced feature points, qualitative inferences can be made regarding the performance of the feature extractor. A powerful feature extractor should learn highly discriminative representations, such that the ideal visualization exhibits clear intra-class clustering and inter-class separation.

As shown in Figure 4, the feature distribution of TFS trained on the few-shot dataset exhibits significant inter-class confusion and intra-class dispersion. Specifically, the cluster boundaries between different categories are blurred and overlapping, while the sample points within the same class are excessively scattered, failing to form compact clusters. As the SNR increases, this phenomenon shows no notable improvement, indicating that the feature extractor fails to learn effective discriminative representations.

In contrast, Figure 5 reveals that the feature distributions of TL and HTL, both pre-trained on the large-scale auxiliary dataset, demonstrate favorable intra-class clustering and inter-class separation. It can be noted that at SNR = −2 dB, the feature distributions of OOK and 8ASK, as well as QPSK and 256QAM, exhibit a certain degree of overlap. However, when the SNR increases to 6 dB, the overlap between OOK and 8ASK is significantly reduced, and the distributions of QPSK and 256QAM show clear separation boundaries. This suggests that as the SNR improves, the features learned by the extractor become more conducive to classification.

#### 5.2.2. Comparison of Classification Accuracy

**Figure 6:**
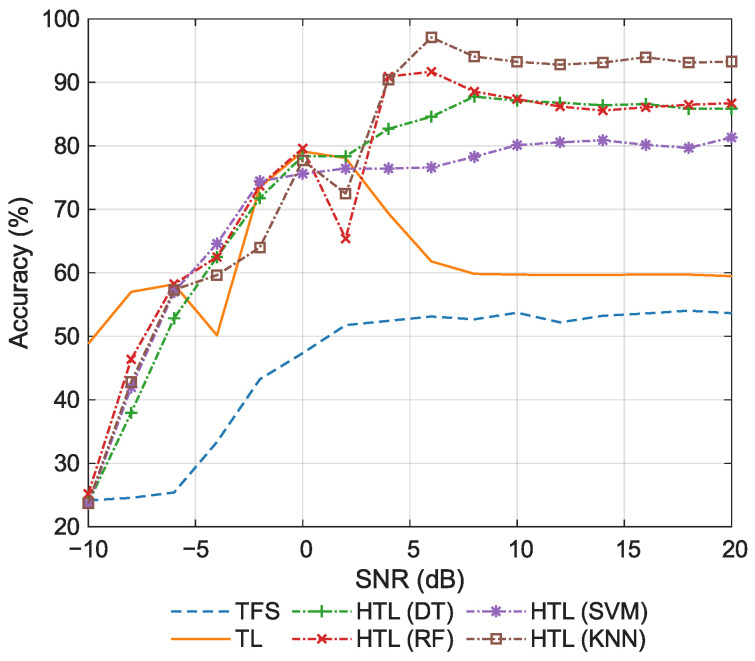
Classification accuracy for the few-shot task.

Then, the classification accuracy of the comparative approaches is evaluated. Figure 6 shows the accuracy at various SNRs. As shown in the figure, when the SNR ranges from −10 dB to −6 dB, TL achieves significantly higher accuracy than the other methods. The primary reason is that under extremely low SNR conditions, the pre-trained features approach white noise. Due to its limited parameter count and the inherited regularization prior from pre-training, the low-dimensional FC head in TL exhibits lower estimation variance than the shallow classifiers used in HTL. As a result, TL maintains a marginal performance advantage over its counterparts even in the few-shot setting at low SNRs. It can also be observed that TL exhibits an inverted U-shaped pattern with respect to SNR, rising initially before declining. When the SNR increases, the discriminative power of features improves, allowing the FC head to converge adequately. However, in the high SNR regime, the feature space becomes more separable, yet the extreme lack of samples causes the model to overfit to spurious correlations, paradoxically reducing test accuracy. Similarly, TFS, which lacks pre-trained priors and is trained with limited samples, is also prone to overfitting, resulting in the lowest accuracy. In contrast, HTL methods that incorporate stabilizing learning objectives or mechanisms exhibit decision boundaries that are less sensitive to few-sample perturbations, thereby keeping variance under control. Consequently, the accuracy of these methods generally increases monotonically with SNR, with only minor local fluctuations due to sampling randomness. Moreover, under high SNR conditions, KNN, in contrast to other ML classifiers, does not require explicit parameter estimation and relies solely on distance-based ranking. This allows it to circumvent decision boundary estimation errors induced by limited samples, resulting in superior generalization capability and consequently higher accuracy.

The average classification accuracy is summarized in Table 5. We focus on the high SNR regime, and the reported value is computed by averaging the accuracy over the 8–20 dB range. The conventional approaches (TFS and TL) achieve relatively low average accuracy (53.30% and 59.69%, respectively). In contrast, all HTL methods significantly outperform them, demonstrating the clear advantage of integrating deep features with traditional ML classifiers. Among the HTL variants, the non-parametric KNN classifier performs best (93.37%). The two tree-based methods (DT and RF) exhibit similar average accuracy (86.62% and 86.71%, respectively), outperforming SVM (80.14%). Overall, the average accuracy decreases in the order: HTL (KNN) > HTL (RF) > HTL (DT) > HTL (SVM) > TL > TFS, indicating that the inherent characteristics of the classifier play a decisive role in the classification performance.

#### 5.2.3. Comparison of Confusion Matrix

**Figure 7:**
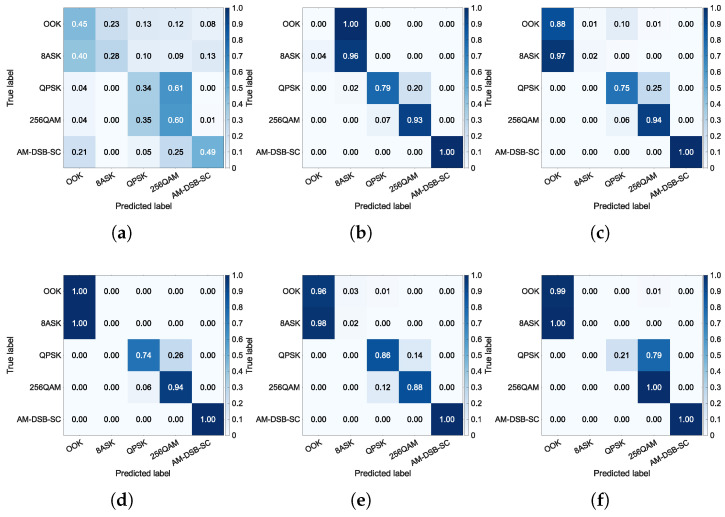
Confusion matrices at SNR=−2 dB. (**a**) TFS. (**b**) TL. (**c**) HTL (DT). (**d**) HTL (RF). (**e**) HTL (SVM). (**f**) HTL (KNN).

**Figure 8:**
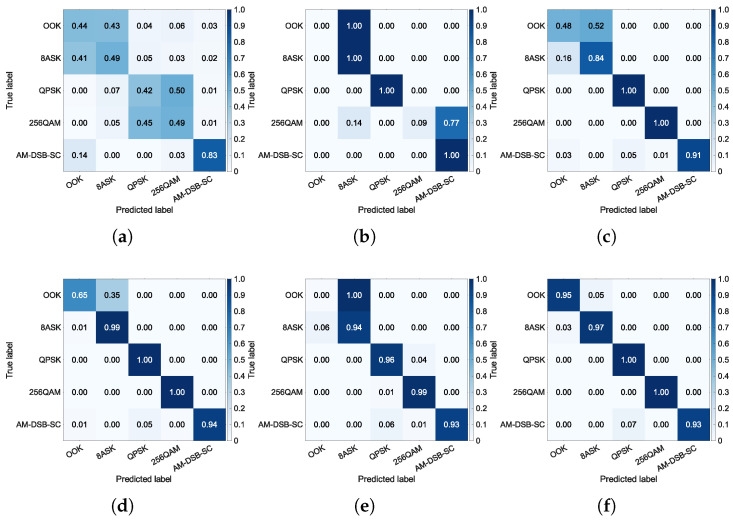
Confusion matrices at SNR=6 dB. (**a**) TFS. (**b**) TL. (**c**) HTL (DT). (**d**) HTL (RF). (**e**) HTL (SVM). (**f**) HTL (KNN).

To gain deeper insight into the performance differences of each method across categories, a confusion matrix analysis is conducted. The confusion matrix visually illustrates the classification accuracy for each class and the confusion patterns between categories, thereby revealing specific class pairs that pose challenges for the model. Through comparison of confusion matrices across different methods and SNRs, the robustness and generalization capability of the models can be more comprehensively evaluated, explaining the root causes of differences in classification accuracy. The confusion matrices at SNR=−2 dB and SNR=6 dB, are shown in Figure 7 and Figure 8, respectively.

Under low SNR conditions, specifically at −2 dB, Figure 7 shows that TFS suffers from substantial error rates, while TL and HTL achieve significantly better category-level accuracy. This demonstrates that the classifier acquired from the auxiliary dataset is beneficial for the few-shot task. This result is corroborated by a comparison of Figure 4 and Figure 5, which reveals that TL and HTL achieve tighter clusters and greater distances between classes than TFS at the same SNR. It can also be noted that all approaches consistently show significant confusion between classes OOK and 8ASK and between classes QPSK and 256QAM. This observation aligns with the overlapping regions of the feature clusters in Figure 4a and Figure 5a, indicating that insufficient separability of feature representations is a common challenge shared by these methods. While inter-class confusion at low SNRs suppresses the performance difference between TL and several HTL variants, this difference will become prominent at higher SNRs as a result of improved feature separability.

Under high SNR conditions, such as 6 dB, Figure 8 demonstrates that TFS still fails to adequately optimize its feature extractor due to the few-shot constraint, resulting in no significant performance improvement. This result is consistent with the blurred inter-class boundaries observed in Figure 4b. Meanwhile, TL shows confusion between classes OOK and 8ASK and between classes 256QAM and AM-DSB-SC, which also corresponds to the feature proximity observed in Figure 5b. In comparison, the HTL variants with DT, RF, or SVM as the classifier successfully resolve the misclassification between classes 256QAM and AM-DSB-SC, with only minor residual errors remaining between classes OOK and 8ASK. This validates that replacing the FC layer with traditional ML classifiers enhances few-shot generalization capability. Notably, HTL with KNN produces a confusion matrix that closely resembles a diagonal identity matrix. This superiority stems from KNN’s distance-based mechanism, which operates without parameter estimation and avoids decision boundary errors caused by limited samples, resulting in high category-level accuracy with minimal inter-class confusion. The aforementioned advantages substantiate the superior performance, reported in Table 5, of the HTL approach when integrated with KNN, which yields the highest classification accuracy.

#### 5.2.4. Comparison of Computational Efficiency

In addition to classification accuracy, the computational efficiency of different methods should also be evaluated for practical applications. To ensure fair comparison, all network modules are executed on a personal computer CPU [Intel(R) Core(TM) i9-13900HX @ 2.20 GHz], providing consistent computational conditions across all evaluated approaches. Based on extensive Monte Carlo trials, a detailed comparison of the inference time performance of different methods in the few-shot setting is summarized in Table 6.

The shared feature extraction backbone establishes a consistent computational baseline across all methods, which constitutes the primary burden compared to the classifiers. Therefore, the dominant cost is attributed to this invariant phase, not the subsequent classification strategy. The classifier exhibits substantial variation in computational requirements. Traditional methods (TFS and TL) achieve a remarkably low inference time of 0.009 ms, benefiting from their simple FC architecture. Among the HTL variants, the tree-based methods show contrasting performance: DT requires only 0.061 ms, while RF demands 2.150 ms, which is the highest among all methods. This is because RF needs to construct multiple DTs to form its ensemble. SVM exhibits comparable efficiency to DT, with an inference time of 0.067 ms. KNN, though considerably slower (0.272 ms) than both SVM and DT, still surpasses RF in speed. The computational overhead of KNN stems from its lack of an explicit model; instead, it must compute distances between each test sample and every sample in the training set. Given the very limited training samples in few-shot tasks, the computational cost of KNN remains manageable, constituting a key advantage in such settings.

It is important to note that these measurements represent CPU-based execution. For real-world deployment, significant acceleration could be achieved through GPU or FPGA implementations, leveraging parallel computing capabilities to further reduce the processing time. The current results demonstrate that even with CPU processing, the total inference time for all methods remains well within practical constraints for real-time applications, provided the signal dwell time exceeds the processing duration. These findings have important implications for practical deployment. The analysis confirms that the proposed HTL framework, particularly with the KNN classifier, not only achieves state-of-the-art classification performance but also maintains competitive computational efficiency, making it well-suited for practical FSMR applications.

### 5.3. Impact of Key Parameters on Performance

This subsection presents a systematic investigation into the impact of two key parameters on classification performance: the training set size and the test sample length. These parameters are selected for their critical role in evaluating model robustness and deployment potential in real-world applications. The analysis of training set size characterizes the relationship between classification accuracy and data volume, reflecting common scenarios where labeled examples are scarce. The examination of test sample length addresses practical challenges in resource-constrained environments, where signals are often truncated or fragmented due to bandwidth limitations or hardware constraints.

#### 5.3.1. Training Set Size

To investigate the impact of training set size on classification performance of different methods in few-shot scenarios, the number of training samples is varied to evaluate its specific effect on classification accuracy. Specifically, building upon the 5-shot setup described in Section 5.1, scenarios with 1- and 20-shot are added, covering a broad range from extremely limited samples to relatively sufficient samples.

**Figure 9:**
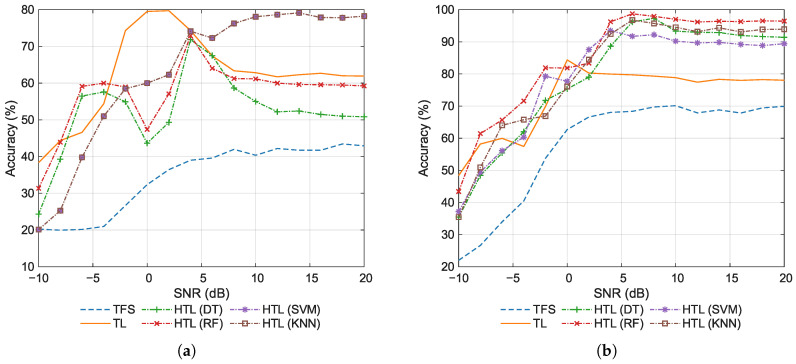
Classification accuracy for various training set sizes. (**a**) 1-shot. (**b**) 20-shot.

Figure 9 shows the classification accuracy at various SNRs for 1-shot and 20-shot scenarios. By comparing Figure 6 and Figure 9, it can be deduced that increasing the number of training samples improves the accuracy, which is expected. Specifically, in the 1-shot scenario, as shown in Figure 9a, HTL with DT or RF exhibits significant accuracy fluctuations as the SNR increases. Moreover, the accuracy of these methods falls below that of TL at high SNRs, indicating their limited performance with only one training sample. Moreover, HTL with KNN achieves a remarkably high accuracy of nearly 80% under high SNR conditions even in the 1-shot scenario. This further validates the effectiveness of employing KNN as the classifier in the HTL approach. It is noteworthy that HTL with either SVM or KNN yields identical classification performance, as both classifiers achieve identical performance in the 1-shot setting. In the 20-shot scenario, as shown in Figure 9b, all HTL methods achieve accuracy around or above 90% at high SNRs. This indicates that all ML classifiers demonstrate strong performance in few-shot scenarios when provided with relatively sufficient training samples. In this case, RF yields the highest accuracy, followed by KNN in second place, and then DT and SVM trailing behind.

The average classification accuracy for 1-shot and 20-shot scenarios is summarized in Table 7. By comparing Table 5 and Table 7, a significant influence of the number of training samples on classification performance can be observed. As the number of training samples increases from 1 to 20, the classification accuracy of all methods shows significant improvement. TFS improves from 42.05% to 69.10%, while TL rises from 62.40% to 78.31%. The HTL methods demonstrate more substantial gains, particularly the tree-based methods: DT increases dramatically from 53.09% to 93.07%, and RF surges from 60.05% to 96.67%. This indicates that tree-based methods exhibit the highest sensitivity to sample size, while conventional TFS and TL methods show relatively lower sensitivity. The sensitivity of KNN and SVM falls between these two extremes. Notably, as an inherently non-parametric method, KNN requires no parameter estimation and exhibits strong robustness, achieving optimal or near-optimal accuracy across various few-shot scenarios with different sample sizes.

#### 5.3.2. Test Sample Length

It is worth noting that in realistic scenarios, the test sample length may deviate from the training sample length. To investigate its influence on classification performance, in addition to the 1024-point length used in Section 5.1, 64- and 256-point lengths are also considered. To adapt to the network’s input requirement, two methods are tested: (1) Zero-padding: shorter sequences are padded with zeros to reach the required 1024-point length. (2) Replication padding: shorter sequences are extended to 1024 points via replication and concatenation.

**Figure 10:**
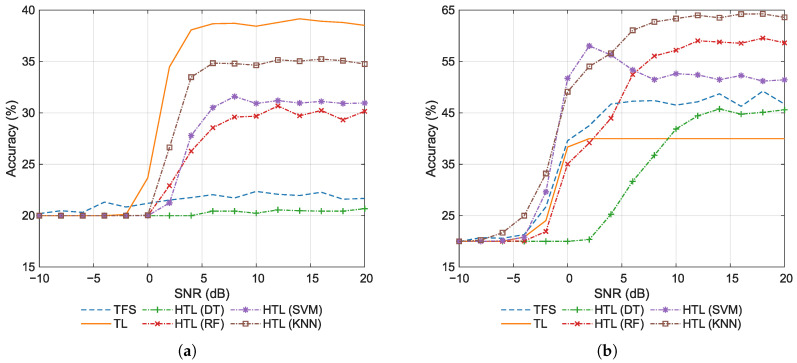
Classification accuracy for various test sample lengths using zero-padding. (**a**) Length 64. (**b**) Length 256.

**Figure 11:**
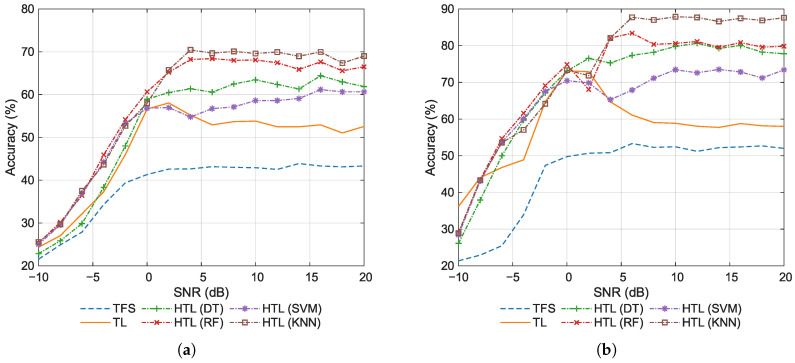
Classification accuracy for various test sample lengths using replication padding. (**a**) Length 64. (**b**) Length 256.

Figure 10 shows the classification accuracy using zero-padding for sample lengths of 64 and 256 points, while Figure 11 shows the counterpart results using replication padding. A comparison of Figure 10 and Figure 11 indicates that the accuracy of all methods is substantially higher using replication padding than using zero-padding. Zero-padding introduces artificial discontinuities (abrupt transitions to zero) at the signal boundaries. Although the network operates directly in the time domain, these discontinuities distort the local context for convolutional filters, effectively injecting task-irrelevant artifacts into the feature maps and compromising their discriminative power. From a signal processing perspective, such time-domain discontinuities correspond to high-frequency spectral leakage, which can spuriously activate the learned frequency-selective filters in the ResNet backbone, thereby contaminating the feature representation. In contrast, replication padding maintains amplitude continuity, preserving the local statistical properties of the signal. This provides a consistent and realistic context for the convolutional filters across the entire input length, enabling them to extract more robust features that are intrinsic to the modulation schemes. Consequently, the model receives a cleaner and more informative feature representation from the outset, leading to superior classification accuracy.

Therefore, based on the preceding analysis, replication padding is recommended over zero-padding for shorter sequences to meet network input requirements, as it more effectively mitigates boundary-induced distortion. In what follows, the results obatained by using replication padding are primarily discussed. By comparing Figure 6 and Figure 11, it can be observed that there is a consistent decline in overall accuracy for all methods as the sample length is reduced from 1024 to 64 points. This indicates that longer sequences provide richer temporal/frequency features for more discriminative classification. Notably, the performance ranking of these methods exhibits stability regardless of the sample length. This finding indicates that methods optimal for long sequences also perform best with short sequences. Consequently, for deployment in resource-constrained or data-fragmented environments, the need for model re-evaluation and re-selection can be eliminated. Compared to other HTL variants, HTL with KNN consistently achieves the highest performance across various sample lengths, demonstrating that its success is attributable not to specific sequence durations but to its inherent generalization capability. This robustness stems from KNN’s instance-based, non-parametric nature, which avoids strong distributional assumptions. Its decisions remain effective with shortened sequences as long as relative feature relationships are preserved in the feature space. These results further prove that HTL with KNN is the most robust method among all HTL variants.

The average classification accuracy for test sample lengths of 64 and 256 points using replication padding is summarized in Table 8. By comparing Table 5 and Table 8, a considerable influence of sample length on classification performance can be noticed. As the sample length decreases from 1024 to 64 points, the classification accuracy of all methods shows a systematic drop. TFS decreases from 53.30% to 43.15%, while TL drops from 59.69% to 52.71%. The HTL methods demonstrate similar trends, with DT decreasing from 86.62% to 62.69%, RF from 86.71% to 67.01%, SVM from 80.14% to 59.41%, and KNN from 93.37% to 69.27%. This indicates that all methods exhibit sensitivity to sample length. Both TFS and TL exhibit only marginal performance degradation, which may indicate suboptimal learning processes or the presence of negative transfer. The KNN variant of HTL maintains the highest accuracy even with shorter sequences, making it a robust choice for few-shot classification.

### 5.4. Evaluation on Other Frameworks and Datasets

This subsection systematically investigates the generalizability and robustness of HTL across diverse experimental configurations. First, we extend the evaluation beyond conventional TL frameworks and study the integration with meta-learning architectures. Second, we evaluate classification performance using an alternative dataset split that differs from the original split employed in earlier sections. These complementary analyses are designed to demonstrate the method’s adaptability to varying learning paradigms and its resilience across different datasets.

#### 5.4.1. Assessment on the Meta-Learning Framework

To further validate the effectiveness of incorporating ML classifiers, we integrate HTL into meta-learning architectures. Similar to TL-based methods, a feature extractor with strong representational capacity can be obtained by meta-learning-based methods. Thus, by using the deep features obtained by the feature extractor, ML classifiers can be trained on top. The prototypical network [44] is adopted as the representative meta-learning framework, and the related processing steps align with the methodology outlined in the previous sections.

**Figure 12:**
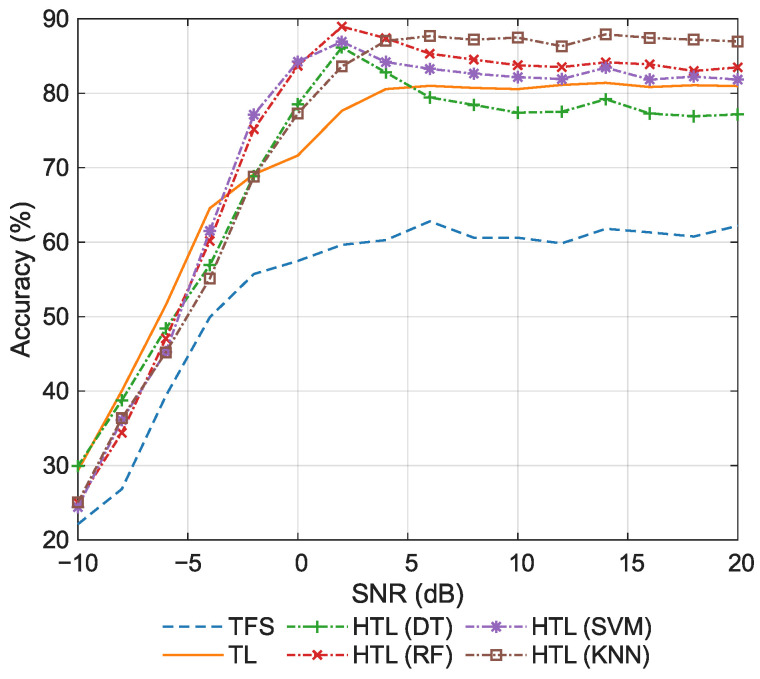
Classification accuracy via the meta-learning framework.

Figure 12 shows the classification accuracy via the meta-learning framework. The superior performance of HTL compared to TFS and TL is once again verified, demonstrating its robustness and adaptability. Furthermore, this result validates the effectiveness of HTL within the meta-learning framework, indicating that its applicability extends beyond specific TL paradigms. Notably, HTL with KNN delivers particularly outstanding performance, achieving significantly higher accuracy across most SNR ranges compared to other HTL variants, as well as traditional baseline methods. This underscores its distinctive advantages in decision boundary optimization. Furthermore, compared with other variants, KNN involves only three principal hyperparameters: the number of neighbors, the distance metric, and the distance weight. Typically, the Euclidean distance is adopted as the metric, thereby leaving only the two hyperparameters listed in Table 4 to be selected. Therefore, KNN demonstrates considerable practical engineering merits due to its model simplicity and parameter efficiency, distinguishing it from other variants that rely on complex architectures or require extensive hyperparameter tuning.

#### 5.4.2. Validation with a Separate Dataset Split

The applicability of HTL is further validated on a different dataset. Unlike the configuration in Table 3, a distinct data split is adopted here: the auxiliary dataset includes OOK, 8ASK, QPSK, 16PSK, 16APSK, 16QAM, 32QAM, 128QAM, and 256QAM, while the few-shot dataset comprises 4ASK, BPSK, 8PSK, AM-SSB-WC, and AM-DSB-WC. This new auxiliary dataset is used for pre-training the feature extractor, while the few-shot dataset is employed to train the entire model (in TFS) or to train only the classifier component (in TL or HTL). Following this setup, recognition performance is evaluated by comparing the classification accuracy of different methods on the few-shot dataset.

**Figure 13:**
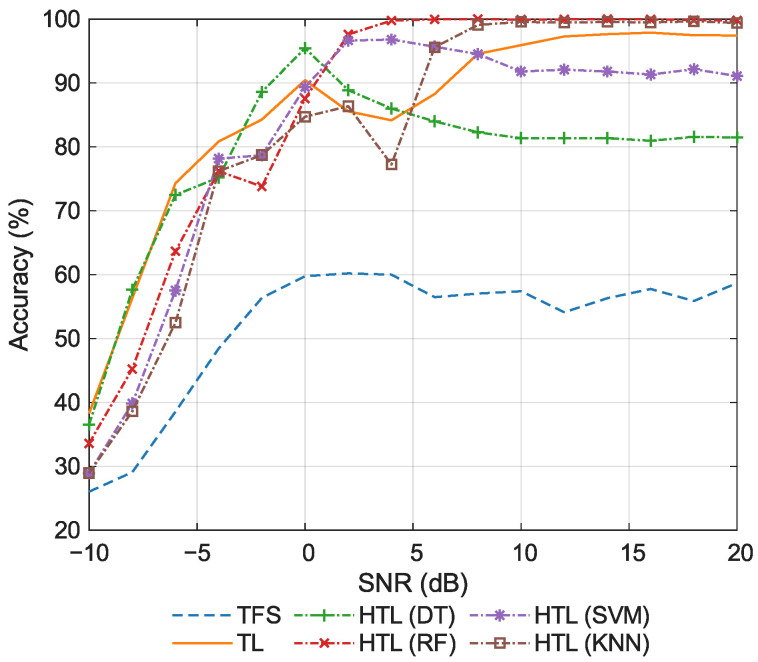
Classification accuracy for a separate dataset split.

Figure 13 shows the classification accuracy for the new few-shot task. The results reconfirm that HTL with KNN has superior accuracy on this new dataset, further validating its decision-making capabilities across diverse data environments. Notably, HTL with RF also achieves competitively high accuracy, demonstrating the effectiveness of the HTL framework. However, the DT and SVM variants of HTL exhibit comparatively unsatisfactory performance, even falling below the conventional TL baseline in certain SNR ranges. This outcome reveals a critical limitation: HTL variants may struggle to maintain consistent generalization across varying experimental conditions when the underlying ML classifier depends on multiple hyperparameters that require careful tuning, such as pruning strategies in DTs or regularization and kernel selection in SVM. The observed performance variability indicates that sensitivity to hyperparameter settings can substantially undermine the robustness and transferability of the method. In contrast, KNN maintains superior and stable accuracy with minimal hyperparameter reliance.

## 6. Conclusions

This study has addressed the critical challenge of modulation recognition in few-shot scenarios where conventional DL approaches face significant limitations. The proposed HTL approach effectively bridges this gap by synergistically combining deep feature extraction with traditional ML classifiers. The experimental investigation has provided substantial evidence supporting the effectiveness and superiority of HTL, especially its KNN variant. Through feature visualization techniques, the superior clustering capability of HTL has been visually confirmed. Comparative analyses verify the method’s robustness and computational efficiency. Additionally, confusion-matrix examinations demonstrate its effectiveness in reducing inter-class misclassification. Sensitivity studies have corroborated the method’s strong adaptability to variations in both training data volume and test sequence duration. Further evaluations conducted across diverse learning frameworks and datasets confirm its strong transferability. These findings carry both theoretical and practical implications. Theoretically, this work challenges the conventional wisdom that DL approaches inherently outperform traditional ML methods. By mitigating the reliance on large labeled datasets, HTL presents a viable and efficient solution for data-scarce real-world tasks such as cognitive radio and spectrum monitoring.

Future efforts will build upon this work along two main directions. First, more advanced network architectures could be explored to further enhance feature extraction capabilities, such as hybrid models combining CNNs with gated RNNs like LSTM or GRU. Second, the core idea of HTL could be integrated with modern learning paradigms, such as self-supervised and semi-supervised learning, to boost its adaptability and generalization in cross-domain scenarios.

## Figures and Tables

**Table 1:** Parameter differences of several ML classifiers.

ML Classifier	Function Space G	Parameter Space Ω
KNN	The functions based on voting or averaging within a local neighborhood.	The entire training dataset.
SVM	Space of linear functions, or linear functions in a high-dimensional feature space defined by a kernel function.	The weight vector and bias of the hyperplane (and the Lagrange multipliers corresponding to support vectors).
DT	All possible piecewise constant functions (defined by a set of if-then rules).	The structure of the tree (including split features, split thresholds, and leaf node values).
RF	All possible combination functions (e.g., voting, averaging) of multiple DT outputs.	The set of structures of multiple DTs.

**Table 2:** Output dimensions of each layer in the backbone network.

Layer	Output Dimension
Input	2×1×1024
Residual Stack	32×1×512
Residual Stack	32×1×256
Residual Stack	32×1×128
Residual Stack	32×1×64
Residual Stack	32×1×32
Residual Stack	32×1×16
FC/SELU	128
FC/SELU	128

**Table 3:** Datasets and parameter settings used in the experiments.

Description	Auxiliary Dataset	Few-Shot Dataset
Modulation classes	4ASK, BPSK, 8PSK, 32PSK, 32APSK, 64APSK, 128APSK, 64QAM, AM-SSB-WC, AM-DSB-WC, FM, GMSK	OOK, 8ASK, QPSK, 256QAM, AM-DSB-SC
SNR range	−10–20 dB (2 dB interval)	−10–20 dB (2 dB interval)
Number of samples (per class)	65,536	65,536
Sample length	1024	1024

**Table 4:** Hyperparameters used by several ML classifiers.

ML Classifier	Hyperparameters
KNN	n_neighbors=1
	weights=‘distance’
SVM	kernel=‘linear’
	class_weight=‘balanced’
DT	max_depth=5
	max_features=‘log2’
RF	max_features=‘log2’

**Table 5:** Average classification accuracy for the few-shot task.

Method	Value
TFS	53.30%
TL	59.69%
HTL (DT)	86.62%
HTL (RF)	86.71%
HTL (SVM)	80.14%
HTL (KNN)	**93.37%**

Note: The highest accuracy is highlighted in bold.

**Table 6:** Inference time for the few-shot task.

Method	Inference Time of Different Model Components (ms)	
	Feature Extractor	Classifier
TFS	5.688	**0.009**
TL		**0.009**
HTL (DT)		0.061
HTL (RF)		2.150
HTL (SVM)		0.067
HTL (KNN)		0.272

Note: The inference time of the feature extractor is identical for all methods. For the classifier component, lower inference time is better, and the minimum value is highlighted in bold.

**Table 7:** Average classification accuracy for various training set sizes.

Method	1-Shot	20-Shot
TFS	42.05%	69.10%
TL	62.40%	78.31%
HTL (DT)	53.09%	93.07%
HTL (RF)	60.05%	**96.67%**
HTL (SVM)	**78.00%**	89.91%
HTL (KNN)	**78.00%**	94.09%

Note: For each scenario (column), the highest accuracy is highlighted in bold.

**Table 8:** Average classification accuracy for various test sample lengths using replication padding.

Method	Length 64	Length 256
TFS	43.15%	52.17%
TL	52.71%	58.37%
HTL (DT)	62.69%	79.14%
HTL (RF)	67.01%	80.27%
HTL (SVM)	59.41%	72.59%
HTL (KNN)	**69.27%**	**87.30%**

Note: For each scenario (column), the highest accuracy is highlighted in bold.

## Data Availability

The dataset used in this study is publicly available at https://www.deepsig.ai/datasets (accessed on 30 November 2025).
